# Tourette syndrome: effects of acetyl-leucine in two individuals

**DOI:** 10.1007/s00415-025-13136-7

**Published:** 2025-05-19

**Authors:** Eva Maria Hofer, Richard Musil, Lennard Strupp, Peter Falkai, Michael Strupp, Katharina A. Schindlbeck

**Affiliations:** 1 Department of Psychiatry and Psychotherapy, LMU University Hospital, LMU Munich, Nussbaumstrasse 7, 80336 Munich, Germany; 2 Oberberg Fachklinik, Bad Tölz, Germany; 3 Faculty of Law (Student), LMU Munich, Munich, Germany; 4 Department of Neurology, LMU University Hospital, LMU Munich, Munich, Germany; 5 Center for Neurosciences, The Feinstein Institutes for Medical Research, Manhasset, NY USA

Dear Sirs,

Tourette syndrome (TS) is a childhood-onset neurodevelopmental disorder, characterized by involuntary movements and vocalizations. The disorder is prevalent into adulthood, disturbing voluntary movements or communication, and often accompanied by behavioral comorbidities. Behavioral therapy approaches are recommended as treatment according to the European guidelines for Tourette Syndrome and tic disorders due to their tolerability and strengthening of the patients’ self-regulatory control [1]. However, local accessibility is very limited in many countries and the feasibility is often restricted due to low introspective ability at a young age or low IQ, or due to low motivation or ability to invest the time and effort required for practicing behavioral therapy [1]. Pharmaceutical treatments in TS include first-, second-, and third-generation antipsychotic agents as well as other agents like tiapride or clonidine. While these pharmaceutical therapies have demonstrated efficacy in treating tics, they are often associated with short- and long-term adverse effects [1]. Therefore, alternative treatments with fewer side effects would represent a major advancement for TS patients.

Acetyl-leucine, a modified amino acid, shows symptomatic and disease-modifying effects in lysosomal storage disorders [2], namely Niemann-Pick disease type C (NPC) [3] and GM2 gangliosidoses [4]. It has recently been approved by the FDA for the treatment of Niemann-Pick disease type C. Several case studies have further reported promising outcomes of AL in conditions such as cerebellar ataxia [5], isolated REM sleep behavior disorder [6], a prodromal condition of α-synucleinopathies, namely Parkinson’s disease, as well as restless legs syndrome [7]. In two individuals with idiopathic REM sleep behavior disorder, long-term treatment with this medication resulted in symptomatic improvement and stabilization or reversal of progression markers assessed by dopamine and metabolic imaging [6]. AL enters enzyme-controlled pathways that correct metabolic dysfunction and improves energy adenosine triphosphate (ATP) production [2, 8]. Given its symptomatic effects in several neurological disorders associated with dopamine-related cerebral dysfunction, we investigated whether acetyl-leucine could also benefit patients with TS.

Three patients that were diagnosed with TS according to DSM-V TR criteria in our outpatient clinic provided written informed consent for off-label therapy under individual case rules in accordance with CARE guidelines and the Declaration of Helsinki. The participants were evaluated at baseline, and follow-up visits at three weeks (FU3), six weeks (FU6) and six weeks post-treatment discontinuation (FU12). The clinical evaluation included the Yale Global Tics Severity Scale (YGTSS), a semi-structured clinical interview to assess the severity of tics with scores ranging from 0 to 100 points. Premonitory sensations were assessed with the Premonitory Urge for Tics Scale (PUTS), a questionnaire with a maximum sum score of 36 points. The Yale-Brown Obsessive Compulsive Scale (YBOCS), a semi-structured clinician-administered interview, was used to assess the severity of obsessions and compulsions with a maximum score of 40. Quality of life was measured with the Gilles de la Tourette Syndrome-Quality of Life scale (GTS-QoL; maximum score 100 points). The YGTSS was used as outcome measure, while the remaining tests were used as secondary measures. Adverse events were investigated at each follow-up visit. The results are presented as individual values; no statistical analysis was performed.

Patient 1 (P1), a 28-year-old male, had suffered from multiple motor tics since the age of 9 and additional vocal tics developing over time. Due to the significant distress caused by the tics, he developed depressive symptoms in early adulthood, which were successfully managed with outpatient psychotherapy. Several pharmacological treatments, including tiapride, aripiprazole, and cannabinoids, were attempted to alleviate his tics but either failed to produce a significant reduction or were discontinued due to intolerable side effects. At the time of the individual treatment with AL, the patient was not actively engaged in psychotherapy or on any medication. Patient 2 (P2), a 40-year-old male, had been suffering from multiple motor tics and throat clearing since the age of 8. At the age of 31, the patient developed torticollis, which was intermittently managed with botulinum toxin. At the time of the treatment, dystonic movements of the neck were part of the motor tic, however, there were no signs of dystonic postures. He also experienced intermittent episodes of anxiety and difficulty falling asleep. At the time of the individual treatment attempt, the patient was on a stable medication consisting of gabapentin (300 mg/d) for trigeminal neuralgia and opipramol (50 mg/d) to manage anxiety symptoms. The patient had not received psychotherapy or medication for his tics. Patient 3, a 20-year-old male, discontinued the treatment after six days due to abdominal discomfort caused by the number of pills taken.

P1 and P2 were treated with AL for about six weeks (5 g/day, a dosage also given in some previous studies [5, 6]). No serious adverse events were observed during the treatment. After six weeks of AL treatment, both patients experienced improvements in tic severity: P1 showed a steady and substantial reduction of tic severity with YGTSS total scores decreasing from 62 at baseline (BL) to 55 at the six-week follow-up (FU6) and further improving to 41 at the twelve-week follow-up (FU12) (see Fig. 1). Patient 2 (P2) also showed an initial improvement, with scores dropping from 77 at BL to 54 at FU6. A slight increase was observed at FU12, where the score rose to 58. Both patients also reported enhanced tic-related quality of life, as measured with the GTS-QoL. Patient 1 showed an improvement in quality of life, with GTS-QoL scores decreasing from 51 at BL to 46 at FU6, and further to 36 at FU12. Patient 2 experienced a similar trend, with scores dropping from 55 at BL to 53 at FU6, and further improving to 36 at FU12. Premonitory urges, measured by the PUTS, exhibited only minor fluctuations: In P1, PUTS scores increased slightly from 26 at BL to 27 at FU6, before decreasing to 23 at FU12. P2, by contrast, showed a gradual increase in PUTS scores from 28 at BL to 29 at FU6, further rising to 32 at FU12. Obsessive–compulsive symptoms, assessed by the YBOCS, also improved in both patients: In P1, YBOCS scores decreased from 17 to 11 at FU6, with a slight increase to 15 at FU12, remaining lower than baseline. In P2, YBOCS scores dropped from 11 at BL to 10 at FU6, and further reduced to 8 at FU12.

**Fig. 1:**
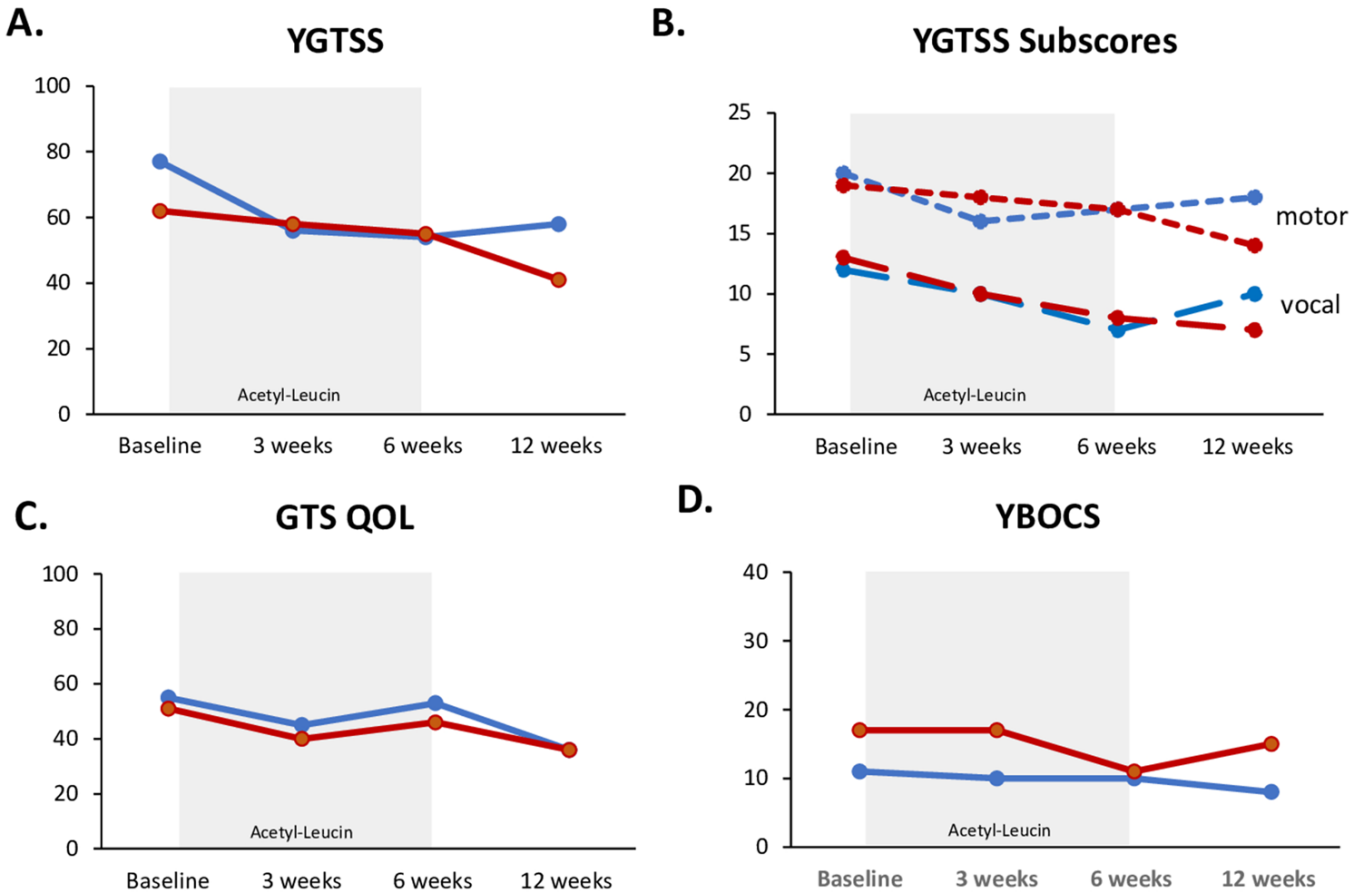
Clinical effects of AL in two TS patients. Clinical assessments of **A** Total Yale Global Tics Severity Scale (YGTSS), **B** YGTSS motor and vocal subscales, **C** Gilles de la Tourette Syndrome-Quality of Life scale (GTS-QoL), and **D** Yale-Brown Obsessive Compulsive Scale (YBOCS) are shown for patient 1 (red line) and patient 2 (blue line) at baseline and during follow-ups at 3, 6, and 12 weeks. The grey bar highlights the six-week therapy period with acetyl-leucine (5 g/day)

The two treated TS patients showed clinically meaningful improvement of tics and tic-related symptoms following short term AL treatment for six weeks. The treatment effect was reflected in three different outcome measures: i) a decline in both motor and vocal tics measured by the YGTSS; ii) a reduction of obsessive–compulsive symptoms measured by the YBOCS; and iii) an improvement of tic-related quality of life indicated by the GTS-QoL. Six weeks after discontinuation of AL we observed continuous effects of tic reduction compared to baseline. An overall 20% reduction in the YGTSS total score in our patients indicates a clinically meaningful improvement in tic severity. Evidence from post hoc analysis of both a psychotherapy study and a pharmaceutical trial in pediatric Tourette Syndrome suggests that a reduction exceeding 20% in the YGTSS total score is predictive of a positive treatment response [9, 10]. In contrast, a recent meta-analysis reported that the placebo response in patients with tic disorders corresponds to a mean decrease of 11 points in the YGTSS total score, equivalent to an 11% reduction of tic severity [11]. The study has several limitations. The treatment period of six weeks is short for the assessment of tics that are characterized by a waxing and waning pattern. Furthermore, we cannot exclude the possibility of a placebo effect, as neither the patients nor the investigators were blinded. In conclusion, the improvement of motor and vocal tics and the absence of severe adverse effects indicate that AL may have therapeutic potential for TS, warranting further investigation in randomized placebo-controlled trials.

## Data Availability

Deidentified data will be made available on reasonable request from interested investigators for the purpose of replicating results.
